# The contributions of proficiency and semantics to the bilingual sentence superiority effect

**DOI:** 10.1017/s1366728922000748

**Published:** 2022-11-28

**Authors:** Portia N. Washington, Robert W. Wiley

**Affiliations:** Department of Psychology, University of North Carolina Greensboro, Greensboro, NC, USA

**Keywords:** shared syntax, sentence processing, sentence superiority effect, bilingual proficiency

## Abstract

A long-standing question about bilingualism concerns which representations are shared across languages. Recent work has revealed a bilingual Sentence Superiority Effect (SSE) among French–English bilinguals reading mixed-language sentences: identification of target words is more accurate in syntactically grammatical than ungrammatical sentences. While this ability to connect words across the two languages has been attributed to a rapid parsing of shared syntactic representations, outstanding questions remain about the role of semantics. Here, we replicate the SSE in Spanish–English bilinguals (e.g., better identification of vacío in “my vaso is vacío” [my glass is empty] than “is vaso my vacío” [is glass my empty]). Importantly, we report evidence that semantics do contribute to word identification, but significantly less than syntax and only in the context of syntactically grammatical sentences. Moreover, the effect is moderated by language proficiency, further constraining the conditions under which shared cross-linguistic representations are rapidly accessed in the bilingual mind.

## Introduction

Bilingual research has been propelled by questions such as whether bilinguals have separate, language-specific systems, and the extent to which they are capable of processing cross-linguistic information in parallel. The separate syntax account states that bilinguals rely on distinct, language-specific properties during language processing. Behavioral and fMRI data from selective impairment and differential recovery of one language in bilingual aphasic participants has been interpreted as evidence in favor of separate processing (Pearce, 2005). More recently, a stronger case has been made for the shared syntax account that states bilinguals are able to access language information cross-linguistically. Evidence from cross-linguistic priming (e.g., Hartsuiker, Pickering & Veltkamp, 2004; Hsin, Legendre & Omaki, 2013), post-cued report paradigms (e.g., Declerck, Wen, Snell, Meade & Grainger, 2020), eye tracking and fMRI (Marian, Spivey & Hirsch, 2003) has all supported the shared syntax account in both sentence production and comprehension.

For example, Hartsuiker et al. (2004) examined the effects of crosslinguistic priming on Spanish–English bilinguals using a picture description task. Prior to this study, work done on shared or separate systems was primarily concerned with lexical or semantic contexts. The participants were asked to describe pictures to a confederate who read from a script (in English) to prime the participants to use either an active or passive phrase in Spanish. The tendency of the participants to use the same type of phrase as the confederate was interpreted as evidence for shared syntax. This seminal paper explains the shared syntax account by claiming that the grammatical hierarchy consists of the lemmas for Spanish–English equivalents attaching to the same category node (e.g., verb, noun), then forming combinatorial nodes to represent sentence structures (e.g., active or passive).

This study also raised the relevance of language proficiency; specifically, the authors emphasized that the bilinguals in their study were moderately-to-highly proficient and lived in a second language-dominant culture, arguing that “at least for these speakers, the advantage of parsimoniously storing a syntactic rule only once outweighs the disadvantage of having to consider alternatives in another language” (Hartsuiker et al., 2004, p. 412). This implies that shared syntax may depend at least in part on the proficiency and linguistic environment in which the bilinguals find themselves. Moreover, others have stressed that bilingual status is not a binary variable (De Bruin, 2019), and a recent meta-analysis of the “bilingual advantage” (Lowe, Cho, Goldsmith & Morton, 2021) argues that proficiency is an underappreciated consideration in bilingual research. It is therefore important to consider that shared syntax may similarly be a question of degree rather than an absolute.

### The bilingual sentence superiority effect

The origins of the sentence superiority effect (SSE) can be traced to the seminal work of Cattel (1886), who first demonstrated the word superiority effect: participants more accurately recall briefly presented letters when they form a word as opposed to a scrambled string of letters. Extended to sentences (Baddeley, Hitch & Allen, 2009; Toyota, 2001), the SSE refers to a participant’s ability to recall a briefly presented target word more often in the context of a grammatical sentence as opposed to an ungrammatical one. With the development of the post-cued partial report method (Reicher, 1969; Wheeler, 1970), researchers used the Rapid Parallel Visual Presentation (RPVP) paradigm to show that whole word identification is active before individual letter identification in sentence reading (McClelland & Rumelhart, 1981). Recent work has used the RPVP paradigm with post-cued partial report to identify a contribution of syntax to the SSE (Snell & Grainger, 2017), with target word identification being greater when embedded in syntactically grammatical as opposed to ungrammatical sentences. This has been interpreted as evidence for rapid parallel processing of parts-of-speech, such that the reader constructs a sentence-level frame that constrains the candidate words, thereby improving word identification. That claim is supported in part by eyetracking methods that demonstrate multiple words are simultaneously processed in reading sufficiently for their syntactic roles to influence behavior. Specifically, Snell, Meeter, and Grainger (2017) used a gaze-contingent boundary paradigm to investigate syntactic ambiguity in parafoveal processing. Participants read sentences that were syntactically congruent, with the parafoveal preview of the target word matching the target’s part-of-speech (e.g., preview “The young horse | *waved* over the fence” for the target “The young horse | *jumped* over the fence”), or incongruent, with the parafoveal preview having a different part-of-speech (e.g., “The young horse | *table* over the fence). Both faster response times and higher accuracy for congruent compared to incongruent sentences provides clear evidence for simultaneous integration of syntactic information from multiple words.

This research has been extended to bilingual contexts, wherein a rapid parsing of mixed-language sentences allows bilingual participants to report a target word in syntactically grammatical sentences more accurately than ungrammatical sentences, first observed in Declerck et al. (2020) in French–English bilinguals. The authors interpreted their results as evidence of cross-linguistic processing of syntactic properties in parallel, which is in support of the shared syntax hypothesis. Effectively ruling out the role of guessing while using post-cued partial report with RPVP, Wen, Snell, and Grainger (2019) presented ERP evidence showing that sentence structure has a significant influence during the early stages of sentence comprehension (within 300 ms), a finding which has been replicated in the context of a grammaticality judgment task (Wen, Mirault & Grainger, 2021). A question remains about the extent to which this effect can be explained by syntax alone, and in particular parts-of-speech. Previous research observing the SSE acknowledges that there may be at least some contribution of semantics because of the likelihood of syntactically grammatical sentences also being semantically interpretable (e.g., Snell & Grainger, 2017). Recently, Massol, Mirault, and Grainger (2021) examined the effect of incorporating semantically uninterpretable sentences to explore the contribution of semantics in the SSE for monolingual French speakers. Although they identified a significant effect of both syntactic grammaticality and semantic interpretability, the SSE effect arising from semantics was much smaller than that arising from syntax. It is currently unknown whether this pattern is also true of bilingual processing of mixed-language sentences.

**Figure 1.**
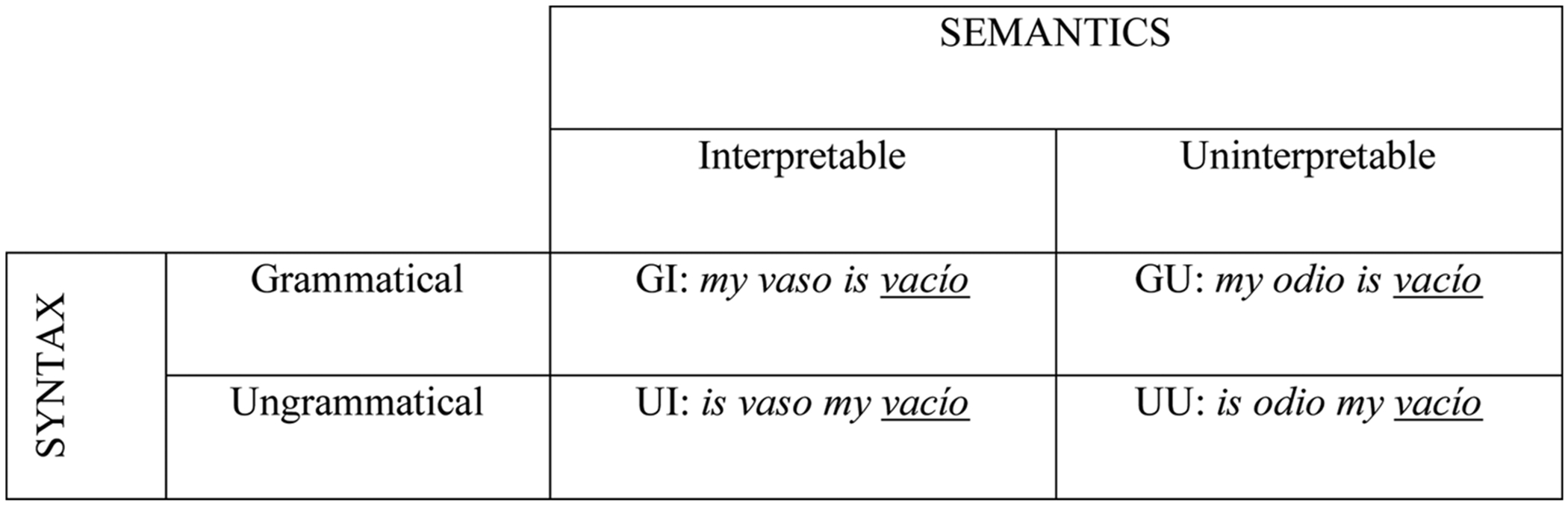
Example of the types of sentences used in the experiments. The target word (vacío) maintains its position in the sentence, while the non-target word subjected to semantic manipulation (vaso/odio) maintains its position, part-of-speech, and word length. GI = Grammatical Syntax, Interpretable Semantics; GU = Grammatical Syntax, Uninterpretable Semantics; UI = Ungrammatical Syntax, Interpretable Semantics; UU = Ungrammatical Syntax, Uninterpretable Semantics.

The current study addresses the following outstanding questions: 1) does the bilingual SSE, first reported in Declerck et al. (2020), replicate in Spanish–English/English–Spanish bilinguals? 2) how does language proficiency moderate the bilingual SSE? and 3) how does semantic interpretability contribute to cross-linguistic processing of syntax in mixed-language sentences? To answer these questions, we first adapted the stimuli and RPVP paradigm used in Declerck et al. (2020) to replicate the bilingual SSE in a different population of bilinguals (Figure 1). We then expanded the stimuli to include semantically uninterpretable sentences, by replacing a single (non-target) word in each sentence while keeping all other factors in the sentence constant (language, parts-of-speech, word lengths, and the target word and its position in the sentence). To anticipate our results, we replicated the bilingual SSE in Spanish–English/English–Spanish bilinguals, demonstrating that this effect of (un)grammatical word order in mixed-language sentences is not specific to French–English, and furthermore we found that this effect is moderated by proficiency. Moreover, we report a significant interaction between syntax and semantics, such that semantic intepretability only moderates the bilingual SSE when the word order is grammatical in both Spanish and English (but not when the word order is ungrammatical in both languages). These results provide novel evidence generally in line with recent work (e.g., Snell & Grainger, 2017; Wen et al., 2019, 2021), supporting the hypothesis that it is primarily information about parts-of-speech that contribute to rapid identification of the words within grammatical sentences. The notable exception is that the role of semantics was found to differ in the bilingual SSE compared to the monolingual SSE, as reported in Massol et al. (2021) – a possible indication that integration of semantic information differs in the case of cross-linguistic processing. We return to these points in the discussion.

## Methods & materials

### Participants

For Experiment 1, data were collected from 57 individuals with 9 excluded from the analyses^1^. This sample size was based on previous work (e.g., Declerck et al., 2020 indicated that as few as N = 24 was sufficient to detect the SSE with the same procedure and number of items). For Experiment 2, data were collected from 153 individuals with 18 excluded from analyses^2^, with no overlap in participants between the two experiments. This sample size was based on a sensitivity analysis (conducted with the R package *simr*, version 1.0.5; Green & MacLeod, 2016) that indicated N = 148 participants would be sufficient to detect an effect size as small as a log odds ratio = 0.04 for the interaction between syntactic and semantic grammaticality, with power = 0.8. Participants were recruited from Amazon Mechanical Turk (MTurk) using CloudResearch (Litman & Robinson, 2020), which is linked to MTurk and provided additional data collection control (i.e., to target participants who speak both English and Spanish). They provided informed consent according to IRB protocols and were compensated $5.00. In addition to professing bilingual status, prior to the main task, the participants completed a version of the Lextale-Esp measure of Spanish proficiency (Izura, Cuetos & Brysbaert, 2014), adapted by the authors for the online software Psytoolkit (Stoet, 2010, 2017). This measure was used to assess the Spanish proficiency of each participant and allowed for investigating an interaction between the bilingual SSE and a range of Spanish proficiency. English proficiency was not assessed, based on the assumption that the participant recruitment pool would provide only highly proficient speakers of English. This assumption was supported empirically by participants’ responses to a language background questionnaire: 96% of the participants reported using English on a daily basis versus just 36% for Spanish, and the mean age of acquisition for English was 1.67 years (median = 0, i.e., from birth), versus 5.21 years for Spanish (median = 1). Tables 1 and 3 summarize the language background responses respectively for Experiment 1 and 2.

### Stimuli

#### Experiment 1

The stimuli from Declerck et al. (2020) were translated to fit our population. We only made changes that fell within the criteria set by Declerck et al., and only when necessary based on the effects of translating the French words to Spanish (e.g., differences in which words are cognates in English). Briefly, each sentence had four words, 2 to 6 letters long, none of which were cognates. Half of the words in the sentence were in English and the other half in Spanish, the order varying randomly to prevent the participant from guessing the language of the target word. The target words were equally divided between English and Spanish. Each of the 200 grammatical sentences had an ungrammatical counterpart that consisted of the same words in a scrambled order (holding the target word in the same position). We refer hereafter to the fully grammatical sentences as GI (Grammatical Syntax, Interpretable Semantics), and to the ungrammatical counterparts with scrambled word order as UI (Ungrammatical Syntax, Interpretable Semantics).

#### Experiment 2

**Figure 2.**
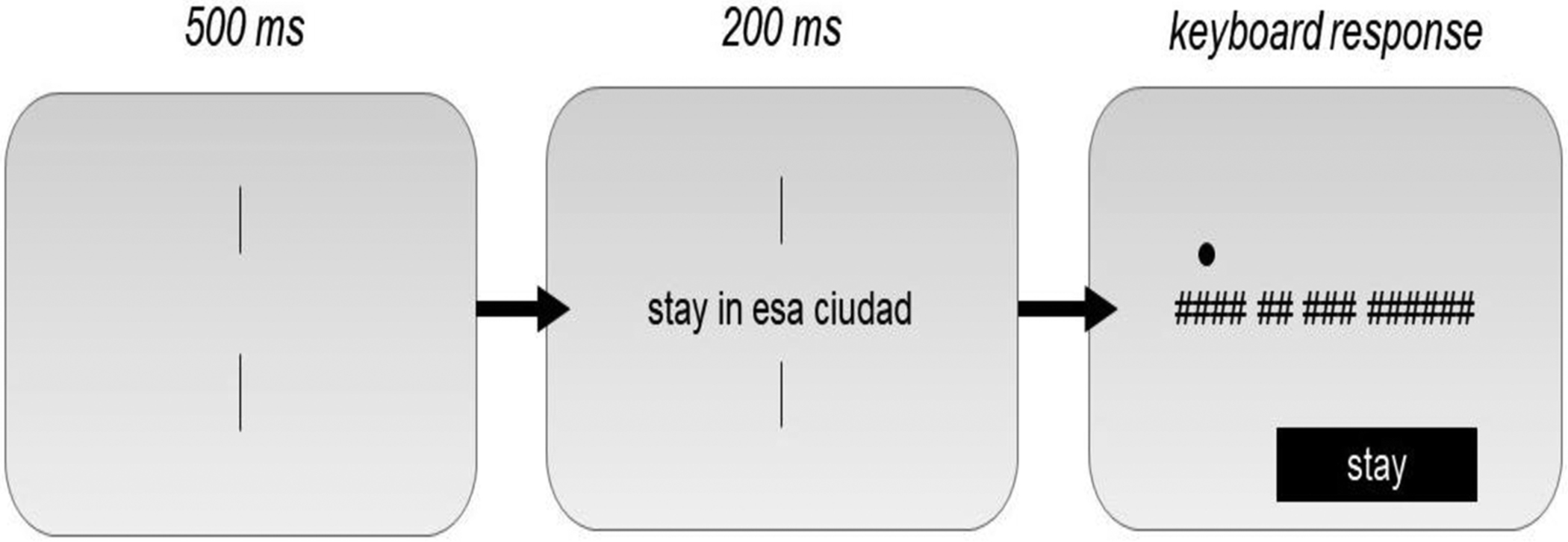
Illustration of the trial sequence for Experiments 1 and 2. In this grammatical sentence, the cued target word is “stay” and can be typed after the hash marks appear.

Semantically uninterpretable versions of the sentences from Experiment 1 were created by replacing a non-target word with a semantically anomalous one, maintaining the same part-of-speech, length, and position. These sentences are hereafter referred to as either GU (Grammatical Syntax, Uninterpretable Semantics) or UU (Ungrammatical Syntax, Uninterpretable Semantics). Seven sentences differed otherwise from those in Experiment 1 (three were changed and four new ones were added)^3^, resulting in a final list of 204 sentences, each with four versions (Figure 2).

#### Interpretability Ratings of Experiment 1 and 2 Sentences

Subsequent to collecting the data for both experiments, a separate group of 80 participants was asked to rate the sentences for their interpretability. These participants were recruited in the same manner as those in Experiments 1 and 2 and were compensated $2.50 for their time. All of the GI sentences as well as their UI and GU counterparts were rated on a 5-point scale, with 1 = Uninterpretable and 5 = Interpretable. Participants rated 102 GI sentences and either 102 UI or 102 GU sentences, and were allowed to freely view the sentences for as long as they wanted (unlike the main experiments, where sentence presentations were 200 ms). Full details about this task are presented in the Supplementary Materials (Supplementary Materials, Section 1: Interpretability Ratings). Summarizing the results, the mean rating for GI sentences was 4.5 (sd = 0.30), UI sentences 2.12 (sd = 0.39), and GU sentences 2.52 (sd = 0.53). This confirmed that the manipulations of both syntax and semantics affected the interpretability of the sentences as intended. However, six of the GI sentences received a median rating below “Somewhat Interpretable”, and an additional two GU sentences failed to be rated as less interpretable than their GI counterparts. We therefore removed those items from analyses, as well as one item from Experiment 1 that was presented to participants with an error. Therefore, 193 items were included in the analyses of Experiment 1 and 196 in the analyses of Experiment 2.

The sentences used for both experiments, their interpretability ratings, and the processed data are all available on the Open Science Foundation (OSF) at: https://osf.io/k5es9/?view_only=d7104e73d7044dc397fc58d498447cab

### Procedure

This study was approved by the authors’ home institution’s IRB. The experiment was programmed with Psytoolkit (Stoet, 2017) and administered online. All participants gave informed consent prior to completing the experiment. The Lextale-Esp proficiency measure was completed first, followed by eight practice trials of the RPVP task. The main experiment consisted of 200 (Experiment 1) or 204 (Experiment 2) Spanish–English trials. Each participant saw 100 grammatical and 100 ungrammatical sentences (Experiment 1), or 51 sentences per combination of syntactic grammaticality and semantic interpretability (Experiment 2). Experiment 1 stimuli were organized into two pairs of counterbalanced lists, such that each participant saw either the grammatical or ungrammatical version of each sentence (but never both). The same was true for Experiment 2, except more lists were needed for counterbalancing due to the 2 × 2 manipulation. The order of sentences within each list was randomized.

The procedure followed that of Declerck et al. (2020): each trial started with two vertical bars at the center of the screen (500 ms), followed by four words between the bars (200 ms), after which each letter was replaced with a hashmark (Figure 2). A cue mark appeared above the target word as well as a place for participants to type their answers; the participant’s screen did not change until they pressed the enter key; they were then given feedback in the form of a green (correct) or red (incorrect) circle.

### Analyses

Accuracy was modeled with generalized (binomial) linear mixed-effects regression in R (R Core Team, 2022), package *lme4* (v 1.1-29; Bates, Maechler, Bolker, Walker, Christensen, Singmann, Dai, Scheipl, Grothendieck, Green, Fox, Bauer & Krivitsky, 2021). All continuous variables were mean-centered and scaled by the standard deviation. P-values for the fixed effects were obtained with the likelihood ratio test (LRT) method provided by the *afex* package (v. 0.27-2, Singmann, Bolker, Westfall, Aust & Ben-Shachar, 2020). In addition, for the main analyses of Experiment 1 and 2 only, 95% confidence intervals were computed with the bootstrap method provided by *lme4*. The R code for these models as well as the convergence checks are available with the other files on OSF.

For Experiment 1, the dependent variable (correct/incorrect) on each trial was regressed on the main variables of interest: Syntactic Grammaticality (coded +1 for GI sentences and −1 UI sentences), Proficiency (Lextale-Esp score), the interaction of Syntactic Grammaticality X Proficiency, and the following control variables: Age (in years), Trial Number, Target Word Position (treated as a categorical variable with 4 levels), and Target Word Frequency. In addition to these fixed effects, random effects were included both by-participant and by-item: random intercepts and random slopes for Syntactic Grammaticality.

For Experiment 2, there was also Semantic Interpretability (coded +1 for GI and UI sentences and −1 for GU and UU sentences) plus its interaction with Syntactic Grammaticality (coded +1 for GI and GU sentences and −1 for UI and UU sentences), both as fixed and random slopes, to test whether semantics moderate the effect of syntactic grammaticality (or vice versa). Finally, the interaction between Proficiency and Semantic Interpretability was also included in Experiment 2, alongside the additional control variable of Non-Target Word Frequency (for the semantically-manipulated word^4^). Word frequencies were obtained from SUBTLEX-ESP (Cuetos, Glez-Nosti, Barbón & Brysbaert, 2011) and SUBTLEX-US (Brysbaert & New, 2009) respectively for Spanish and English words.

#### A note about model (non-)convergence

The mixed-effects models of both Experiments 1 and 2 encountered a model convergence warning, relating specifically to the estimation of the correlation between the random slope for Syntactic Grammaticality and/or Semantic Interpretability by-participants, on the one hand, and the random intercept by-participants on the other. We followed the suggestions outlined in Brauer and Curtin (2018) and Bates et al. (2021) to investigate this issue. We determined that the warning was due to too little variance remaining in the effect of grammaticality across participants, once also controlling for the interaction between grammaticality and proficiency. Nonetheless, we determined via the allFit function in the *lme4* package that the fixed-effects estimates were unaffected by this issue (i.e., all optimizers estimated the same coefficients for the fixed effects to at least the third decimal, and dropping the by-participants random slope while maintaining the by-item random slope similarly had a negligeable effect on the estimates). We therefore report the results of the models despite the convergence “warnings”, consistent with the recommendations of the authors of the *lme4* package (Bates et al., 2021), and the fact that the inferences drawn in this manuscript are based only on the fixed-effects estimates (see Brauer & Curtin, 2018; Singmann & Kellen, 2019, for more support and discussion of this strategy).

## Results

The final sample of 48 participants included 12 compound bilinguals (learned both Spanish and English from birth), 19 English–Spanish bilinguals (English from birth and Spanish on average from age 11, sd = 5 years), 12 Spanish–English bilinguals (Spanish from birth and English on average from age 6, sd = 2 years), and 5 Other (some language besides English or Spanish from birth; English on average from age 4, sd = 4 years, and Spanish also from age 4, sd = 4 years). The average score on the Spanish proficiency test, Lextale-Esp, was 0.69, sd = 0.15. The participants were asked how frequently they communicate in each language (1 = daily, 2 = most days, 3 = occasionally, 4 = rarely); for English, the average was 1.04, sd = 0.2, and for Spanish, the average was 1.88, sd = 0.8. This information is summarized in Table 1.

### Experiment 1

**Figure 3.**
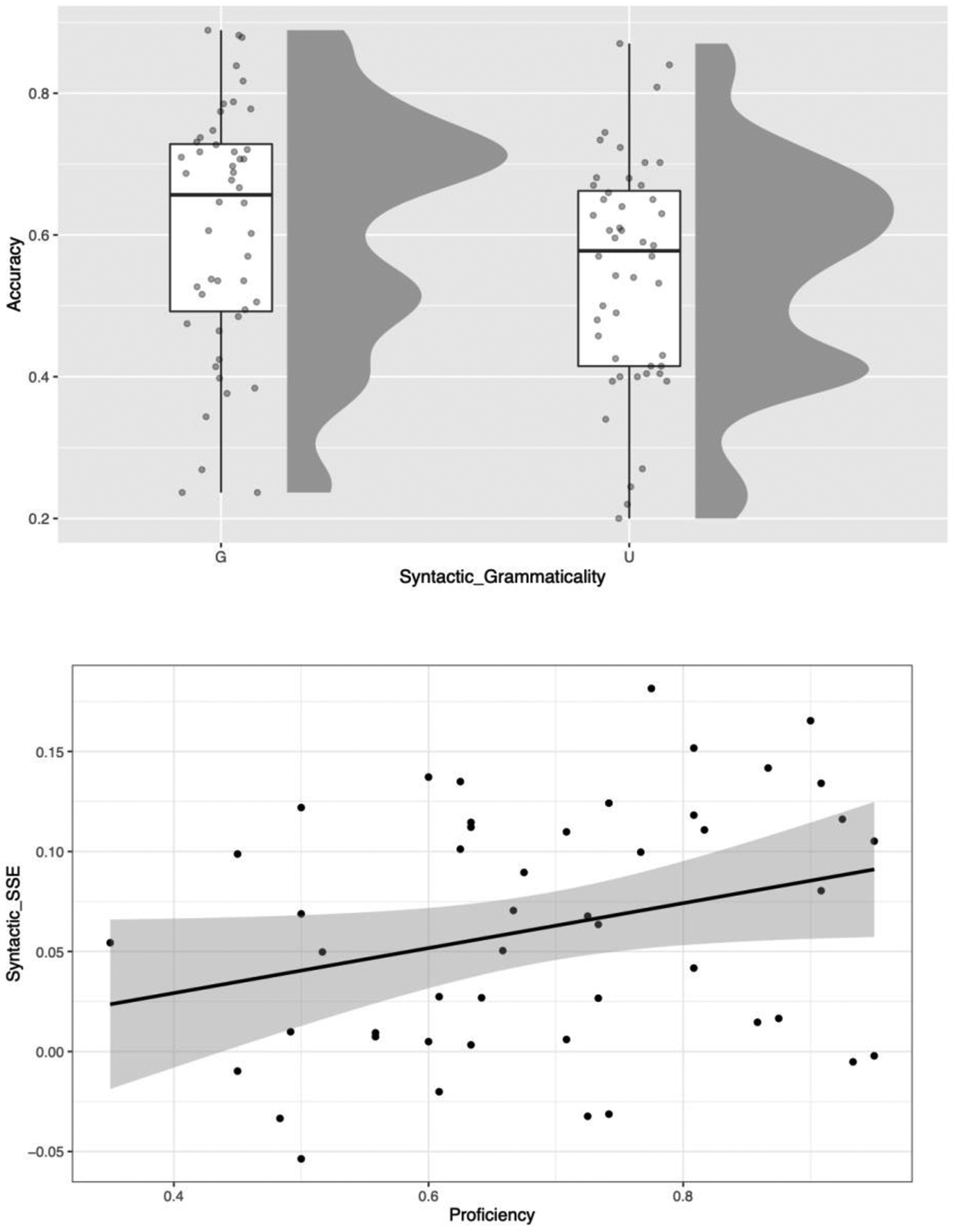
Results of Experiment 1. *Top panel*: raw accuracy, by participants, for sentences with grammatical syntax (G) versus ungrammatical syntax (U). The box plots depict the median and interquartile ranges, with each dot representing a single participant. *Bottom* panel: the sentence superiority effect (SSE), depicted as mean accuracy on grammatical - ungrammatical sentences (y-axis), is moderated by Spanish proficiency (x-axis), with a larger SSE for higher-proficiency versus lower-proficiency individuals. The line depicts the positive linear trend, with each dot representing a single participant. The gray region reflects standard error of the mean.

**Figure 4.**
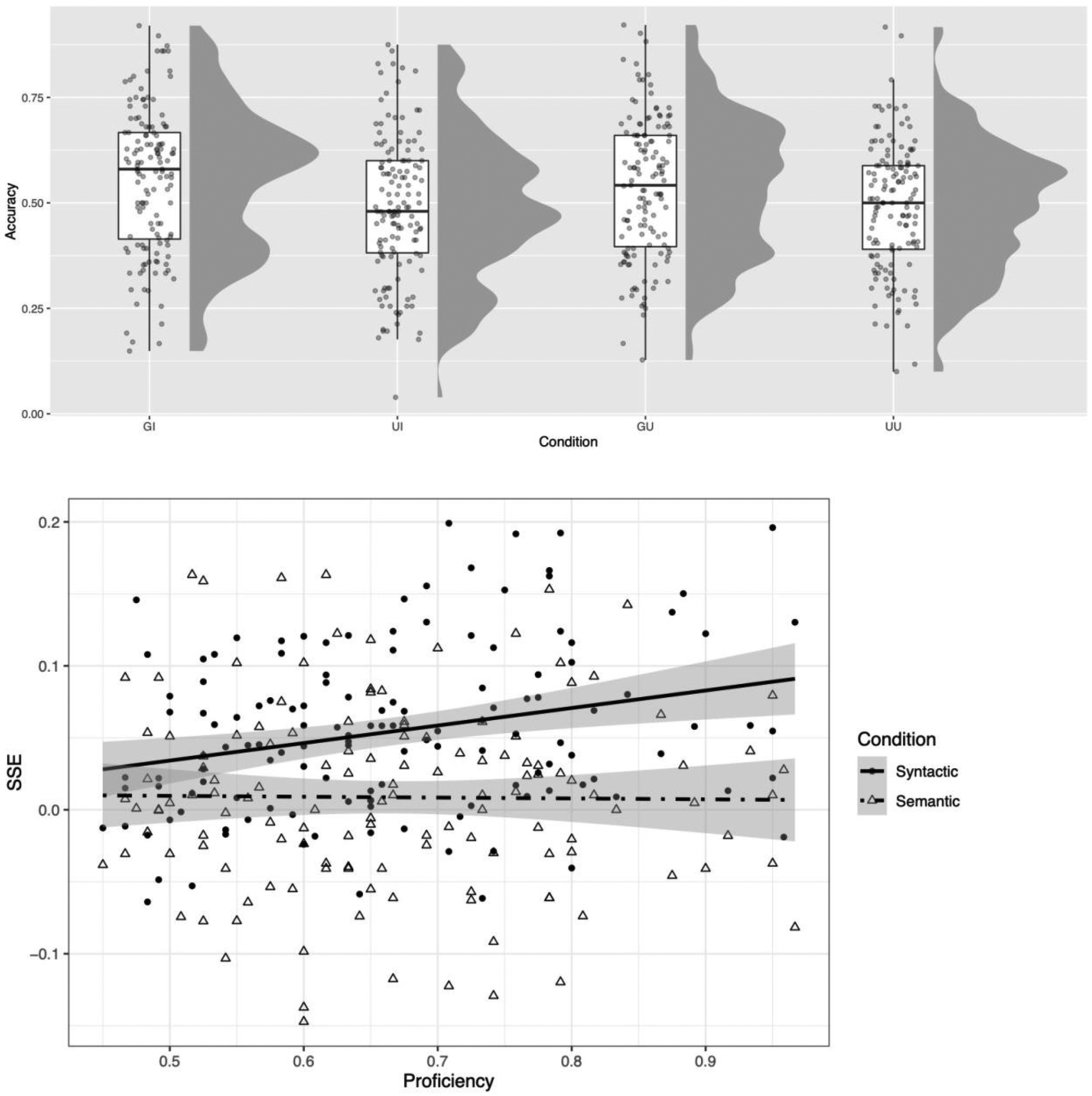
Results of Experiment 2. Top panel: GI = Grammatical Syntax, Interpretable Semantics; UI = Ungrammatical Syntax, Interpretable Semantics; GU = Grammatical Syntax, Uninterpretable Semantics; UU = Ungrammatical Syntax, Uninterpretable Semantics. The box plots depict the median and interquartile ranges, with each dot representing a single participant. *Bottom* panel: the sentence superiority effect (SSE), depicted for Syntax as mean accuracy on GI/GU-UI/UU sentences (circles/solid line), and for Semantics as mean accuracy on GI/UI-GU/UU sentences (triangles/dashed line). The effect of Syntax is moderated by Spanish proficiency (x-axis), with a larger SSE for higher-proficiency versus lower-proficiency individuals, as shown in the positive linear trend (solid line). There is no such moderation for the effect of Semantics (dashed line). The gray region reflects standard error of the mean.

The raw data, aggregated by participant, are depicted in Figure 3 (see Supplementary Materials Appendix, Figure A1, for the data aggregated by item). The fixed effects from the regression model are summarized in Table 2 (the full model including the random effects is reported in the Appendix, Table A1). Depicted as raw averages by participant (Figure 3 top), the main effect of Syntactic Grammaticality was significant, estimated to be a log odds ratio (OR) = 0.19, 95% CI [0.09, 0.28] (likelihood ratio test, LRT, p-value < 0.001). The estimated marginal means were obtained with the R package *emmeans* (version 1.5.5-1; Lenth, 2021), which revealed that accuracy was 8.4% lower for target word identification in syntactically ungrammatical compared to grammatical sentences (71.2% versus 62.8%); this is comparable to the effect size reported for French–English bilinguals in Declerck et al. (2020) of 7.3%. The SSE was significantly moderated by Spanish Proficiency (Figure 4 bottom), estimated log OR = 0.06, 95% CI [0.01, 0.12] (p ≈ 0.017). This can be interpreted as a larger effect of proficiency on identifying targets in grammatical sentences compared to ungrammatical ones. Follow-up comparisons indicated a significant effect of Spanish Proficiency for grammatical sentences (log OR = 0.37, p ≈ 0.017), but only a marginal effect for ungrammatical sentences (log OR = 0.27, p ≈ 0.059).

To address the possibility that the effect of Grammaticality was driven not by cross-linguistic processes, but rather by rapid translation of the mixed-language sentences into monolinguals ones (e.g., “she está with ellas” → “she is with them”), an analysis was conducted by coding each error as a translation-equivalent (e.g., target word = “guantes”, response = “gloves”) or not. On average, just 3.2% of participants’ errors were translation-equivalents (range across participants: 0%–16.2%, *sd* = 2.8%)^5^. A generalized linear-mixed effects model was used to analyze just the incorrect trials, with identical structure to the one reported for the main analyses, and the dependent variable indicating translation-equivalent errors (1) versus all other errors (0). Neither Syntactic Grammaticality (log OR = 0.04, p ≈ 0.95), Spanish Proficiency (log OR = −0.09, p ≈ 0.65), nor their interaction (log OR = −0.02, p ≈ 0.87) were significantly associated with rates of translation-equivalent errors. These results are consistent with findings reported by Declerck et al. (2020) that just 1.7% of errors were translation-equivalents, although in that study a statistical analysis was not conducted. The only significant predictors of translationequivalent errors were Trial Order (log OR = −0.41, p ≈ 0.01), indicating that participants made fewer of these errors later in the experiment, and Target Frequency (log OR = 1.01, p ≈ 0.001), indicating that higher frequency words were more prone to this type of error. This model is reported in detail in the Supplementary Materials Appendix (Table A2).

### Experiment 2

The final sample of 135 participants included 42 compound bilinguals (learned both Spanish and English from birth), 51 English–Spanish bilinguals (English from birth and Spanish on average from age 12, sd = 7 years), 23 Spanish–English bilinguals (Spanish from birth and English on average from age 6, sd = 5 years), and 19 Other (some language besides English or Spanish from birth; English on average from age 6, sd = 4 years, and Spanish from age 6, sd = 6 years). The average score on the Spanish proficiency test, Lextale-Esp, was 0.67, sd = 0.13. The participants were asked how frequently they communicate in each language (1 = daily, 2 = most days, 3 = occasionally, 4 = rarely); for English, the average was 1.04, sd = 0.2, and for Spanish, the average was 2.01, sd = 1.0. This information is summarized in Table 3.

The raw data are depicted in Figure 4 (aggregated by participant; see Supplementary Materials Appendix, Figure A2 for aggregation by item), and the fixed effects from the regression model are summarized in Table 4 (the full model including the random effects is reported in the Supplementary Materials Appendix, Table A3). As in Experiment 1, the main effect of Syntactic Grammaticality was significant, log OR = 0.15, 95% CI [0.08, 0.22] (p < 0.001), The interaction of Syntactic Grammaticality X Proficiency was also significant, log OR = 0.041, 95% CI [0.01, 0.09] (p ≈ 0.004), indicating a larger effect of grammaticality for more-proficient participants. There was no significant main effect of Semantic Interpretability, log OR = 0.02, 95% CI [−0.03, 0.07]; similarly, the interaction of Semantic Interpretability X Proficiency was not significant, log OR = 0.002, 95% CI [−0.03, 0.03] (p ≈ 0.894).

However, there was a significant interaction of Syntactic Grammaticality X Semantic Interpretability, log OR = 0.03, 95% CI [0.003, 0,06] (p ≈ 0.032). The estimated marginal means reveal the following pattern of results: for sentences with interpretable semantics, the effect of Syntactic Grammaticality was significant, estimated as 9.0% lower accuracy for ungrammatical sentences, p < 0.001 (Figure 4 top, GI versus UI). For sentences with uninterpretable semantics, the effect of Syntactic Grammaticality was significantly smaller ( p ≈ 0.032), but still itself significant, estimated as 5.8%, p ≈ 0.002 (Figure 4 top, GU versus UU). The results can also be interpreted this way: for sentences with grammatical syntax, the effect of Semantic Interpretability was marginally significant, estimated as 2.7% lower accuracy for uninterpretable sentences, p ≈ 0.055 (Figure 4 top, GI versus GU). For sentences with *ungrammatical* syntax, there was a nonsignificant trend in the opposite direction, 0.05% percent higher accuracy for uninterpretable sentences (p ≈ 0.76).

### Exploratory analyses of Experiments 1 and 2

Two sets of exploratory analyses were conducted on the data from both experiments – full details are reported in the Supplementary Materials available on OSF. Briefly, we assessed whether the results differed between English and Spanish target words (Supplementary Materials Section 2: Analyses of Target Language), and, for sentences with Spanish target words, whether those with diacritic marks differed from those without (Section 3: Analyses of Diacritic Marks). For each of these sets of analyses, we controlled for a familywise error rate of testing each hypothesis twice (i.e., in Experiments 1 and 2) with alpha of 0.05/2 = 0.25 (see, e.g., Rubin, 2017).

#### Target Language

Declerck et al. (2020) demonstrated that the SSE effect in French–English bilinguals was independent of the target language, i.e., there was no significant difference between the magnitude of the SSE assessed by French target words compared to English target words. Similarly, our analyses revealed no significant main effect of Target Language nor an interaction with Syntactic Grammaticality in Experiment, although the trend was for better identification of English words. For Experiment 2, the main effect was significant, with better identification of English target words than Spanish, but there was no interaction with either Syntactic Grammaticality or Semantic Interpretability. The general tendency for better identification of English words plausibly is related to higher proficiency with English relative to Spanish (which may also explain why the numerical trend in Declerk et al. favored French over English, as those participants were L1 French, L2 English); because we did not measure English proficiency, we cannot directly assess this possibility.

#### Diacritic Marks

The final set of exploratory analyses addressed the possibility that diacritic marks, which were present only on (some of) the Spanish words, could contribute to word identification, due to their visual saliency (e.g., Perea, Baciero & Marcet, 2021). We investigated this possibility by re-analyzing the results just for the sentences with a Spanish target word, comparing those with and without diacritic marks (e.g., í, ñ, ó). In total there were nine such targets (less than 5% of all trials). Numerically, words with diacritics were more accurately identified than those without, but this was not significant. Moreover, the diacritic marks did not significantly interact with Spanish Proficiency, the Syntactic Grammaticality effect, or the Semantic Interpretability effect, or Spanish Proficiency.

## Discussion

The present study was designed to address three questions, motivated by the consensus of recent work observing an SSE in both monolinguals and bilinguals and related to the claim that the sentence superiority effect (SSE) is driven by parts-of-speech. First, we aimed to understand how this effect interacts with proficiency, and to replicate the effect in a population of individuals who speak both Spanish and English, as previously it has been demonstrated only in highly proficient French–English bilinguals. Experiment 1 did replicate the bilingual SSE, in a heterogenous population of individuals who speak both Spanish and English. Furthermore, we found the effect to be moderated by the participants’ Spanish proficiency, with the largest effect observed in the most proficient participants. In fact, while there was a clear and significant advantage of having higher proficiency when identifying words in grammatical sentences (p ≈ 0.02), there was relatively weak evidence for an advantage when identifying words in ungrammatical sentences (p ≈ 0.06). This, coupled with the fact that the interaction between proficiency and grammaticality remained when controlling for word frequency, strongly supports the hypothesis that the SSE is driven by expert linguistic knowledge of sentence structures, and not simply greater familiarity with individual words (or domain general processes that might be deployed even by individuals with little or no proficiency).

Experiment 2 expanded the stimuli to include manipulations of semantic interpretability. Our experimental design allowed us to isolate the contribution of semantics from syntactic grammaticality, as well as to test for interactions between each of these with language proficiency. We again replicated the effect of grammaticality as well as the interaction with proficiency. However, syntactic grammaticality and semantic interpretability showed different patterns of results. Our manipulation of the semantic interpretability of a non-target word (e.g., “my vaso is vacío” versus “my odio is vacío”, target word “vacío”) had only a modest effect on identification of the target word in sentences with grammatical syntax (2.7% lower accuracy, marginally significant at p ≈ 0.055). Crucially, however, that small effect was itself significantly different (p ≈ 0.032) in comparison to what we observed in sentences with ungrammatical syntax, where there was a numeric trend in the opposite direction (0.5% higher accuracy) that was nowhere near statistical significance (p ≈ 0.76). In addition, a separate group of participants corroborated that our manipulation of the non-target words did indeed render the sentences less interpretable, rating them on average 2 points less interpretable on a 5-point scale (see Supplementary Materials Section 1: Interpretability Ratings). In sum, the effect of semantics was smaller than that of syntax, was only present in the context of sentences with grammatical word order, and was not moderated by language proficiency.

These results are in line with other recent findings and are interpreted to be in support of the shared syntax account. By observing a significant difference in the post-cued partial report between syntactically grammatical and ungrammatical mixed-language sentences, it can be concluded that the participants completed a rapid parse of words in a sentence, cross-linguistically, in parallel. The fact that semantics were found to have a significant effect on the bilingual SSE indicates that parts-of speech do not fully account for the effect, meaning that the rapid parse of sentence processing includes (at least some) information beyond parts-of-speech, as argued by Massol and colleagues (2021) in the monolingual case. In that work, the authors reported a significant effect of semantic interpretability even in syntactically ungrammatical contexts; however, we found that target words were less-well identified in semantically ungrammatical compared to grammatical sentences only when the word order was grammatical. While these two studies are the only ones to our knowledge to attempt to distinguish between syntactic and semantic contributions to the SSE, this difference in the results suggests that semantic representations may affect cross-linguistic sentence processing only after the syntactic parsing is successful (e.g., recognition of grammatical word order may be a prerequisite for further processing at the level of semantics) – whereas monolinguistic sentences may allow for a contribution of semantics even within syntactically ungrammatical contexts. This hypothesis is supported by the work of Hagoort (2003), which demonstrated an asymmetry in the (simultaneous) processing of syntactic and semantic violations, such that a “syntactic boost” is observed when a semantic violation is put in a grammatically correct sentence. The author did not observe a concomitant semantic boost, which is analogous to our findings here that the effect of semantic grammaticality is observed only when no syntactic violations are present.

Finally, the interaction of proficiency with the effect of syntactic grammaticality but not semantic interpretability further suggests that the simultaneous processing of shared, cross-linguistic syntactic representations requires considerable skill with both languages. The fact that we did not observe any interaction between proficiency and semantics is not surprising given two considerations: first, that models of bilingualism generally posit wholly shared semantic representations (e.g., BiLex – Peñaloza, Grasemann, Dekhtyar, Miikkulainen & Kiran, 2019); and second, we controlled for both target and non-target word frequency statistically in the model, which contributed to accounting for differences in the participants’ knowledge of individuals Spanish words (on the assumption that lower-proficiency individuals would be especially unfamiliar with low-frequency words). Alternatively, it may be that the heterogeneity of our bilingual sample, not only in terms of their Spanish proficiency but also their linguistic background (e.g., compound bilingual, multilingual, etc.), had an impact on our results that we cannot fully assess.

In fact, the exploratory analyses of target language and diacritic marks suggest some future directions for further investigating the factors supporting parallel processing of bilingual (i.e., mixed-language) sentences. For example, the significant effects of proficiency we found suggest that future work should consider planned comparisons between subpopulations of bilinguals, and should assess the proficiency level of both L1 and L2. In addition, the analyses of diacritic marks provided some evidence of differential processing for Spanish words with compared to without accent marks, even though power to detect any such effects was low given the small number of items with accent marks. Moreover, because diacritic marks are not part of English orthography, they might have contributed to overall differences between the target languages. One future direction would assess how these visual-orthographic cues contribute to bilingual syntactic processing, especially in mixed-language sentences. For example, the role that diacritic marks play in reading has been posited to be either linguistic (e.g., Chetail & Boursain, 2019) or visual-orthographic in nature (e.g., Kinoshita, Yu, Verdonschot & Norris, 2021). Research with bilingual populations may provide new insights into critical questions about the status of diacritic marks, considering that their status varies across languages (e.g., lexical stress in Spanish versus vowel quality in German; Perea, Labusch & Marcet, 2022;) as well as within languages (e.g., Spanish accented “él” [he] provides lexical contrast with unaccented “el” [the], whereas accented “marrón” only indicates stress).

## Conclusion

Altogether, these findings have implications for future research on the parallel and integrated nature of bilingual syntax processing. First, this effect has now been observed in French, Spanish, and English, but previous studies have indicated that non-alphabetic scripts may rely more on semantics during the initial parse (Asano & Yokosawa, 2011) – as such, more populations of bilinguals should be explored, including those with non-alphabetic scripts. Indeed, one limitation of this study is that the participants were highly heterogeneous, not only in their Spanish proficiency but also in terms of which language was L1, which language is currently their dominant language, etc.; any of these variables may affect how syntactic or semantic processes contribute to comprehension of the mixed-language sentences. Second, the novel finding of null effects of semantics in the context of ungrammatical syntax (as well as the non-significant interaction between semantics and proficiency) raises a number of questions for future work, including whether the results of Massol et al. (2021) with monolingual participants differ from those reported here due to a difference in mixed-language versus monolingual sentences. Lastly, by identifying a robust effect of syntax on the bilingual SSE, future research should explore the nature of the contribution of parts-of-speech to the rapid parse and the conditions under which semantic representations do or do not contribute – for example, whether certain parts-of-speech are identified during the rapid parse more than others (i.e., nouns and verbs as opposed to articles).

## Supplementary Material

Supplementary Material

## Figures and Tables

**Table 1.** Descriptive statistics for the participants included in Experiment 1. AoA = Age of acquisition (years). Comm. = Communication (1 = daily, 2 = most days, 3 = occasionally, 4 = rarely).

L1	*N*	Age		Spanish Proficiency		English AoA		Spanish AoA		English Comm. Frequency		Spanish Comm. Frequency	
		M	*sd*	M	*sd*	M	*sd*	M	*sd*	M	*sd*	M	*sd*
Compound	*12*	30.33	*8.00*	0.69	*0.13*	0.00	*0.00*	0.00	*0.00*	1.00	*0.00*	1.67	*0.65*
English	*19*	32.11	*6.77*	0.61	*0.13*	0.00	*0.00*	10.95	*5.26*	1.00	*0.00*	2.21	*0.85*
Spanish	*12*	33.42	*9.06*	0.81	*0.11*	5.50	*2.28*	0.00	*0.00*	1.00	*0.00*	1.58	*0.67*
Other	*5*	35.80	*15.61*	0.73	*0.21*	4.40	*4.56*	4.20	*3.56*	1.40	*0.55*	1.80	*0.84*
**Grand Mean**	***48***	**32.38**	***8.67***	**0.69**	***0.15***	**1.83**	***3.06***	**4.77**	***6.23***	**1.04**	***0.20***	**1.88**	***0.79***

**Table 2.** Summary of the mixed-effects model for Experiment 1. The primary variables of interest are highlighted in gray. Log OR = log odds ratio. Confidence intervals obtained by bootstrap method and p-values reflect likelihood ratio tests (LRT). Further details about the model are presented in the Appendix, Table A1. Predictors significant at p < 0.05 in **bold**.

Predictors	Log OR	CI	df	Chisq	p
(Intercept)	−1.13	−1.58 - −0.71			
Target Position [2 vs 1]	2.38	1.88 – 2.89	*3*		**<0.001**
Target Position [3 vs 1]	2.07	1.59 - 2.57			
Target Position [4 vs 1]	1.86	1.38 - 2.41			
Age	−0.29	−0.55 - −0.02	*1*		**0.038**
Trial Order	0.07	0.002 - 0.13	*1*		**0.029**
Target Frequency	0.27	0.11 - 0.45	*1*		**0.003**
Syntactic Grammaticality	0.19	0.09 - 0.28	*1*		**<0.001**
Spanish Proficiency	0.31	0.03 - 0.59	*1*		**0.025**
Syntactic Grammaticality * Proficiency	0.06	0.01 - 0.12	*1*		**0.017**

**Table 3.** Descriptive statistics for the participants included in Experiment 2. AoA = Age of acquisition (years). Comm. = Communication (1 = daily, 2 = most days, 3 = occasionally, 4 = rarely).

L1	*N*	Age		Spanish Proficiency		English AoA		Spanish AoA		English Comm. Frequency		Spanish Comm. Frequency	
		M	*sd*	M	*sd*	M	*sd*	M	*sd*	M	*sd*	M	*sd*
Compound	*42*	33.83	*8.85*	0.65	*0.11*	0.00	*0.00*	0.00	*0.00*	1.05	*0.31*	1.81	*0.92*
English	*51*	35.04	*10.42*	0.65	*0.13*	0.00	*0.00*	12.33	*6.86*	1.02	*0.14*	2.41	*0.98*
Spanish	*23*	31.22	*7.83*	0.75	*0.13*	5.96	*5.29*	0.00	*0.00*	1.09	*0.29*	1.39	*0.66*
Other	*19*	37.32	*12.31*	0.63	*0.10*	5.53	*3.72*	6.16	*5.55*	1.00	*0.00*	2.21	*1.18*
**Grand Mean**	***135***	**34.33**	***9.90***	**0.67**	***0.13***	**1.79**	***3.69***	**5.53**	***7.36***	**1.04**	***0.23***	**2.02**	***1.01***

**Table 4.** Summary of the mixed-effects model for Experiment 2. The primary variables of interest are highlighted in gray. Log OR = log odds ratio. Confidence intervals obtained by bootstrap method and p-values reflect likelihood ratio tests (LRT). Further details about the model are presented in the Appendix, Table A3. Predictors significant at p < 0.05 in **bold**.

Predictors	Log OR	CI	df	Chisq	p
(Intercept)	−1.20	−1.51 - −0.88	*3*	*56*	**<0.001**
Target Position [2 vs 1]	1.83	1.45 – 2.22			**<0.001**
Target Position [3 vs 1]	1.82	1.43 – 2.19			**<0.001**
Target Position [4 vs 1]	1.39	0.95 - 1.82			**<0.001**
Age	−0.19	−0.34 - −0.05	*1*		**0.009**
Trial Order	0.06	0.02 - 0.10	*1*		**0.001**
Target Frequency	0.21	0.05 - 0.35	*1*		**0.005**
Nontarget Frequency	−0.05	−0.18 - 0.08	*1*		0.420
Syntactic Grammaticality	0.15	0.08 - 0.22	*1*		**<0.001**
Semantic Interpretability	0.02	−0.03 - 0.07	*1*		0.350
Spanish Proficiency	0.11	−0.04 - 0.26	*1*		0.121
Syntactic Grammaticality * Semantic Interpretability	0.03	0.003 - 0.06	*1*		**0.032**
Syntactic Grammaticality * Spanish Proficiency	0.04	0.01 - 0.08	*1*		**0.004**
Semantic Interpretability * Proficiency	0.002	−0.03 - 0.03	*1*		0.894

## Data Availability

The data that support the findings of this study, as well as the analysis codes, are openly available from the Open Science Foundation (OSF) at https://osf.io/k5es9/?view_only=d7104e73d7044dc397fc58d498447cab

## Footnotes

1 2 for reporting a diagnosis of dyslexia, 1 for not completing the Spanish proficiency task, and 6 for not completing the experimental RPVP task.

2 2 for reporting a diagnosis of dyslexia, 2 for not completing the Spanish proficiency task, and 14 for not completing the experimental RPVP task.

3 For example, the sentence in Experiment 1 “she está with ellas” (she is with them), target word ellas, has no possible semantically ungrammatical version that maintains the parts-of-speech and word length of a non-target word (e.g., any other pronoun besides “she” would either remain semantically grammatical or render the sentence syntactically ungrammatical).

4 This was done to control for the fact that the mean frequency of the non-target words in the Semantically Interpretable sentences (706 appearances per million) was significantly higher than their counterparts in the Semantically Uninterpretable sentences (237 per million), t(203) = 5.26, p < 0.001.

5 Fully 34% of the translation-equivalent errors were for the pair is/es, which happen to be both orthographically and phonologically very similar in English/Spanish. The rate was almost identical in Experiment 2: 35%.
