# Supplementary material for: The contributions of proficiency and semantics to the bilingual sentence superiority effect
Source: Biling (Camb Engl). Author manuscript; Available in PMC 2025 Apr 21. (PMC12010924; doi:10.1017/s1366728922000748)
Supplement: Supplementary Material [file NIHMS2071580-supplement-Supplementary_Material.pdf]

Supplementary Materials

Section 1: Interpretability Ratings

Participants

80 Participants were recruited from Amazon Mechanical Turk (MTurk) using CloudResearch (Litman & Robinson, 2020), which is linked to MTurk and provided additional data collection control (i.e., to target Spanish-English bilingual participants). All participants professed proficiency in both English and Spanish. They provided informed consent according to IRB protocol and were compensated $2.50.

Stimuli

All of the grammatical sentences from Experiments 1 and 2, as well as the syntactically ungrammatical and semantically uninterpretable counterparts, were randomly divided across four lists. Each list contained an equal number of fully grammatical (Grammatical Syntax, Interpretable Semantics; GI) and either Ungrammatical Syntax, Interpretable Semantics (UI) or Grammatical Syntax, Uninterpretable Semantics (GU) sentences. The fully ungrammatical sentences (Ungrammatical Syntax, Uninterpretable Semantics, UU) were not presented.

Sentences were presented in pairs, one below the other, with the grammatical and either ungrammatical or uninterpretable counterparts randomly labeled as either Sentence A or Sentence B. The order of presentation was fully randomized for each participant.

Procedure

Each participant was asked to provide interpretability ratings for just one of the four lists, containing 208 sentences. After providing consent and attesting to their bilingual status, they were instructed to rate the sentences on a 5-point scale of interpretability. The labels for the scale were Uninterpretable, Somewhat Uninterpretable, Neutral, Somewhat Interpretable, and Interpretable. They were provided four example sentences, all in English, to demonstrate use of the scale. They then completed four practice trials, also all in English, before rating the 208 mixed-language sentences.

Data Processing

For one sentence, five ratings were excluded because they were presented to participants with a typographic error. In addition, 15 participants were entirely excluded for data quality control—specifically, three were excluded for giving all sentences the same rating, and 12 were excluded because they completed the task in an impossibly short amount of time (two seconds or less to read and rate each sentence). The final ratings were therefore based on 28 to 38 responses for GI sentences and 14 to 19 responses for UI and GU sentences.

Summary of Results

The file Interpretability Ratings.xlsx on the Open Science Foundation (https://osf.io/k5es9/?view_only=d7104e73d7044dc397fc58d498447cab) presents each sentence in a row, with columns containing the mean and median ratings for each. A value of 1 was assigned to the rating of Uninterpretable, up to a value of 5 for a rating of Interpretable. A separate sheet is used for each of the three types of sentence (GI, UI, and GU). The mean rating for GI sentences was 4.5 (sd = 0.30), UI sentences 2.12 (sd = 0.39), and GU sentences 2.52 (sd = 0.53).

Section 2: Analyses of Target Language

Exploratory analyses were conducted with respect to the language of the target word (Spanish or English), for both Experiments 1 and 2. The same mixed-effects models as reported in the manuscript had added to them a regressor for "Target Language" (coded +1 for English and -1 for Spanish), as well as interactions between it and Proficiency, Syntactic Grammaticality (for both Experiment 1 and 2) and Semantic Interpretability (for Experiment 2 only). The models were fit using R software (R Core Team, 2022), package *lme4*, version 1.1-29 (Bates et al., 2015). P-values were obtained with the likelihood ratio test (LRT) method provided by the package *afex*, version 0.27-2 (Singmann et al., 2022). The alpha criterion for significant was set to 0.05/2 = 0.025, to account for the familywise error rate of testing the exploratory hypothesis that target language affects the results in two experiments (see, e.g., Rubin, 2017).

Summary of Results

These analyses revealed a non-significant main effect of Target Language: in Experiment 1, such that English words were identified better than Spanish words (log odds ratio, OR = 0.16, $p \approx 0.053$). In Experiment 2, this effect was significant and of slightly higher magnitude (log OR = 0.2, $p \approx 0.005$). Importantly, there was no significant interaction with Syntactic Grammaticality in either Experiment 1 or 2 (uncorrected p's ≈ 0.04 and 0.39). Likewise, there was no significant interaction with Semantic Interpretability in Experiment 2 ($p \approx 0.43$). The trend toward better identification of English target words in general may relate to the fact that the participants were more proficient in English than in Spanish; although we did not explicitly measure English proficiency, on average age of acquisition was younger for English than Spanish and frequency of daily communication was higher for English than Spanish.

Table S2.1. Summary of mixed-effects model analyzing effect of target language on target identification in Experiment 1. Bolded p-values indicate significance at alpha = 0.025.

Formula = Accuracy ~ Target_Position + Age + Trial Order + log(Target_Frequency) + Syntactic_Grammaticality * Spanish_Proficiency + Target_Language + Target_Language:Syntactic Grammaticality + (1 + Syntactic_Grammaticality * Target_Language | Participant) + (1 + Syntactic_Grammaticality | Item)

Predictors	*Log OR*	*Df*	*Chisq*	*p*
(Intercept)	-1.14			
Target Position [2 vs 1]	2.41	*3*	*87.69*	**<0.001**
Target Position [3 vs 1]	2.09			
Target Position [4 vs 1]	1.87			
Age	-0.29	*1*	*3.90*	0.048
Trial Order	0.07	*1*	*4.94*	0.026
Target Frequency	0.27	*1*	*8.44*	**0.004**

Syntactic Grammaticality	0.19	*1*	*14.58*	**<0.001**
Spanish Proficiency	0.24	*1*	*1.58*	0.209
Target Language	0.16	*1*	*3.07*	0.080
Syntactic Grammaticality * Proficiency	0.05	*1*	*2.03*	0.155
Syntactic Grammaticality * Target Language	-0.10	*1*	*4.22*	0.040
				**Random Effects**
σ^2				3.29
τ_{00} Item				1.08
τ_{00} Participant				0.89
τ_{11} Item.Syntactic Grammaticality				0.29
τ_{11} Participant.Syntactic Grammaticality				0.01
τ_{11} Participant.Target Language				0.01
τ_{11} Participant.Syntactic Grammaticality:Target Language				0.00
ρ_{01} Item				-0.06
ρ_{01} Participant.Syntactic Grammaticality				0.50
ρ_{01} Participant.Target Language				-0.14
ρ_{01} Participant.Syntactic Grammaticality:Target Language				-0.31
N Participant				48
N Item				193
Observations				9264
AIC/BIC				9674 / 9809

Table S2.2. Summary of mixed-effects model analyzing effect of target language on target identification in Experiment 2. Bold p-values indicate significance at alpha = 0.025.

Formula = Accuracy ~ Target_Position + Age + Trial Order + log(Target_Frequency) + (Syntactic_Grammaticality + Semantic_Interpretability) * Spanish_Proficiency + Syntactic_Grammaticality:Semantic_Interpretability + Target_Language + Target_Language:(Syntactic Grammaticality + Semantic_Interpretability) + (1 + Syntactic_Grammaticality + Semantic_Interpretability + Target_Language | Participant) + (1 + Syntactic_Grammaticality + Semantic_Interpretability | Item)

Predictors	*Log OR*	*Df*	*Chisq*	*p*
(Intercept)	-1.18			
Target Position [2 vs 1]	1.83	*3*	*87.29*	**<0.001**
Target Position [3 vs 1]	1.79			
Target Position [4 vs 1]	1.36			
Age	-0.20	*1*	*6.70*	**0.010**
Trial Order	0.06	*1*	*10.76*	**0.001**
Target Frequency	0.20	*1*	*7.68*	**0.006**
Nontarget Frequency	-0.05	*1*	*0.48*	0.489
Syntactic Grammaticality	0.15	*1*	*17.72*	**<0.001**
Semantic Interpretability	0.02	*1*	*0.85*	0.372
Spanish Proficiency	0.10	*1*	*2.30*	0.208
Target Language	0.20	*1*	*8.65*	**0.005**
Syntactic Grammaticality * Semantic Interpretability	0.03	*1*	*4.42*	0.038
Syntactic Grammaticality * Spanish Proficiency	0.04	*1*	*8.25*	**0.023**
Semantic Interpretability * Spanish Proficiency	-0.002	*1*	*0.02*	0.901
Syntactic Grammaticality * Target Language	-0.03	*1*	*0.73*	0.393
Semantic Interpretability * Target Language	-0.02	*1*	*0.63*	0.429
		**Random Effects**		
σ^2			3.29	
τ_{00} Participant			0.80	

τ_{00} Item	0.72
τ_{11} Participant.Syntactic_Grammaticality	0.00
τ_{11} Participant.Semantic_Interpretability	0.00
τ_{11} Participant.Target Language	0.04
τ_{11} Item.Syntactic_Grammaticality	0.19
τ_{11} Item.Semantic_Interpretability	0.08
ρ_{01} Participant.Syntactic_Grammaticality	0.45
ρ_{01} Participant.Semantic_Interpretability	0.94
ρ_{01} Participant.Target Language	-0.05
ρ_{01} Item.Syntactic_Grammaticality	-0.12
ρ_{01} Item.Semantic_Interpretability	-0.04
$N_{Participant}$	135
N_{Item}	196
Observations	26442
AIC / BIC	28861 / 29131

Section 3: Analyses of Diacritic Marks

Exploratory analyses were conducted with respect to diacritic marks that appeared on 9 of the target Spanish words (≈ 5% of all the Spanish target words), in consideration of the possibility that diacritic marks both are salient visual clues that are known to affect processing (e.g., Perea et al., 2021). To explore the possibility that diacritic marks may have contributed to the apparent SES, we tested whether the 9 Spanish target words that included a diacritic mark (marrón, vacío, lémur, tú, día, días, aquí, él, and están) were more or less well identified than the non-diacritic mark targets. The same mixed-effects models as reported in the manuscript had added to them a regressor for "Diacritics" (coded +1 for diacritic present and -1 for not), as well as interactions between it and Proficiency, Syntactic Grammaticality (for both Experiment 1 and 2) and Semantic Interpretability (for Experiment 2 only).

Note that these analyses were restricted to trials with a Spanish target, as diacritic marks are not used in English. The models were fit using R software (R Core Team, 2022), package lme4, version 1.1-29 (Bates et al., 2015). P-values were obtained with the likelihood ratio test (LRT) method provided by the package *afex*, version 1.1-1 (Singmann et al., 2022).

Summary of Results

Numerically, words with diacritics marks were better identified than those without; however, the main effects and interactions were not significant in either Experiment 1 or 2 (p's > 0.05, uncorrected likelihood ratio tests). Full details of the mixed-effects models follow below in Tables S3.1 and S3.2.

Table S3.1. Summary of mixed-effects model analyzing effect of diacritic marks on target identification in Experiment 1. Bold p-values indicate significance at alpha = 0.025.

Formula = Accuracy ~ Target_Position + Age + Trial Order + log(Target_Frequency) + Syntactic_Grammaticality * Spanish_Proficiency * Diacritics + (1 + Syntactic_Grammaticality * Diacritics | Participant) + (1 + Syntactic_Grammaticality | Item)

Predictors	*Log OR*	*Df*	*Chisq*	*p*
(Intercept)	-1.01			
Target Position [2 vs 1]	2.63	*3*	*54.29*	**<0.001**
Target Position [3 vs 1]	2.07			
Target Position [4 vs 1]	1.54			
Age	-0.20	*1*	*1.84*	0.176
Trial Order	0.09	*1*	*4.29*	0.038
Target Frequency	0.39	*1*	*9.04*	**0.003**
Syntactic Grammaticality	0.43	*1*	*15.69*	**<0.001**

Spanish Proficiency	0.54	*1*	*10.03*	**0.002**
Diacritics	0.32	*1*	*2.94*	0.086
Syntactic Grammaticality * Spanish Proficiency	0.08	*1*	*1.62*	0.204
Syntactic Grammaticality * Diacritics	0.17	*1*	*2.62*	0.106
Spanish Proficiency * Diacritics	0.06	*1*	*0.80*	0.372
Syntactic Grammaticality * Proficiency * Diacritics	-0.01	*1*	*0.01*	0.914

	**Random Effects**
σ^2	3.29
τ_{00} Item	0.94
τ_{00} Participant	0.96
τ_{11} Item.Syntactic_Grammaticality	0.20
τ_{11} Participant.Syntactic_Grammaticality	0.00
τ_{11} Participant.Diacritics	0.00
τ_{11} Participant.Syntactic_Grammaticality:Diacritics	0.01
ρ_{01} Item	-0.07
ρ_{01} Participant.Syntactic Grammaticality	-0.92
ρ_{01} Participant.Diacritics	0.41
ρ_{01} Participant.Syntactic Grammaticality:Diacritics	-0.99
N Participant	48
N Item	98
Observations	4728
AIC/BIC	4991 / 5166

Table S3.2. Summary of mixed-effects model analyzing effect of diacritic marks on target identification in Experiment 2. Bold p-values indicate significance at alpha = 0.025.

Formula = Accuracy ~ Target_Position + Age + Trial Order + log(Target_Frequency) + (Syntactic_Grammaticality + Semantic_Interpretability) * Spanish_Proficiency + Syntactic_Grammaticality:Semantic_Interpretability + Diacritics * (Syntactic Grammaticality * Semantic_Interpretability + Spanish_Proficiency) + (1 + Diacritics * (Syntactic_Grammaticality + Semantic_Interpretability) | Participant) + (1 + Syntactic_Grammaticality + Semantic_Interpretability | Item)

Predictors	*Log OR*	*Df*	*Chisq*	*p*
(Intercept)	-1.14			
Target Position [2 vs 1]	1.85	*3*	*54.37*	**<0.001**
Target Position [3 vs 1]	1.71			
Target Position [4 vs 1]	0.97			
Age	-0.20	*1*	*6.58*	**0.010**
Trial Order	0.04	*1*	*1.94*	0.164
Target Frequency	0.32	*1*	*10.34*	**0.001**
Syntactic Grammaticality	0.22	*1*	*6.16*	**0.013**
Semantic Interpretability	-0.03	*1*	*0.21*	0.646
Spanish Proficiency	0.21	*1*	*6.23*	**0.013**
Diacritics	0.18	*1*	*1.38*	0.239
Syntactic Grammaticality * Semantic Interpretability	0.07	*1*	*4.42*	0.036
Syntactic Grammaticality * Spanish Proficiency	0.05	*1*	*5.55*	**0.019**
Semantic Interpretability * Spanish Proficiency	-0.02	*1*	*1.01*	0.315
Syntactic Grammaticality * Diacritics	0.04	*1*	*0.25*	0.619
Semantic Interpretability * Diacritics	-0.08	*1*	*1.90*	0.168
Spanish Proficiency * Diacritics	0.02	*1*	*0.49*	0.482
Syntactic * Semantic * Diacritics	0.01	*1*	*0.08*	0.775
		**Random Effects**		
σ^2			3.29	

τ_{00} Participant	0.79
τ_{00} Item	0.68
τ_{11} Participant.Diacritics	0.00
τ_{11} Participant.Syntactic_Grammaticality	0.01
τ_{11} Participant.Semantic_Interpretability	0.00
τ_{11} Participant.Diacritics:Syntactic_Grammaticality	0.01
τ_{11} Participant.Diacritics:Semantic_Interpretability	0.00
τ_{11} Item.Syntactic_Grammaticality	0.20
τ_{11} Item.Semantic_Interpretability	0.07
ρ_{01} Participant.Diacritics	0.59
ρ_{01} Participant.Syntactic_Grammaticality	0.80
ρ_{01} Participant.Semantic_Interpretability	0.67
ρ_{01} Participant.Diacritics:Syntactic_Grammaticality	0.88
ρ_{01} Participant.Diacritics:Semantic_Interpretability	0.09
ρ_{01} Item.Syntactic_Grammaticality	-0.07
ρ_{01} Item.Semantic_Interpretability	0.09
N Participant	135
N Item	99
Observations	13347
AIC / BIC	14763 / 15101

Appendix

Table A1. Summary of mixed-effects model analyzing effect of Syntactic Grammaticality on target identification in Experiment 1. Log OR = log odds ratio. Predictors significant at $p < 0.05$ in bold. AIC/BIC = Akaike Information Criterion/Bayesian Information Criterion.

Formula = Accuracy ~ Target_Position + Age + Trial Order + log(Target_Frequency) + Syntactic_Grammaticality * Spanish_Proficiency + (1 + Syntactic_Grammaticality | Participant) + (1 + Syntactic_Grammaticality | Item)

Predictors	*Log OR*	*CI*	*df*	*Chisq*	*p*
(Intercept)	-1.13	-1.58 – -0.71			
Target Position [2 vs 1]	2.38	1.88 – 2.89	*3*		
Target Position [3 vs 1]	2.07	1.59 – 2.57			

Target Position [4 vs 1]	1.86	1.38 – 2.41		
Age	-0.29	-0.55 – -0.02	*1*	0.038
Trial Order	0.07	0.002 – 0.13	*1*	0.029
Target Frequency	0.27	0.11 – 0.45	*1*	0.003
Syntactic Grammaticality	0.19	0.09 – 0.28	*1*	<0.001
Spanish Proficiency	0.31	0.03 – 0.59	*1*	0.025
Syntactic Grammaticality * Proficiency	0.06	0.01 – 0.12	*1*	0.017
		Random Effects		
σ^2			3.29	
τ_{00} Item			1.07	
τ_{00} Participant			0.85	
τ_{11} Item.Syntactic Grammaticality			0.29	
τ_{11} Participant.Syntactic Grammaticality			0.00	
ρ_{01} Item			-0.08	
ρ_{01} Participant			1.00	
N Participant			48	
N Item			193	
Observations			9264	
AIC / BIC			9734 / 9849	

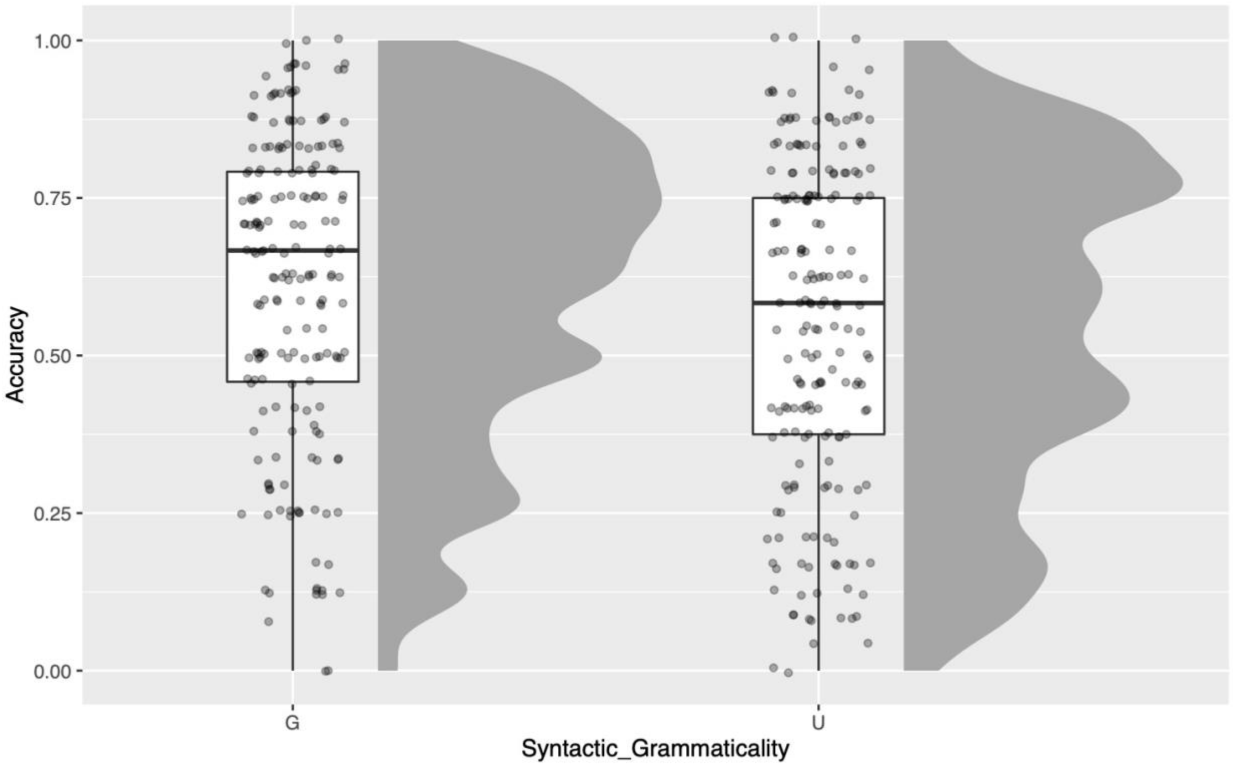

Figure A1. Results of Experiment 1, raw accuracy, by items, for sentences with grammatical syntax (G) versus ungrammatical syntax (U). The box plots depict the median and interquartile ranges, with each dot representing a single item.

Table A2. Summary of mixed-effects model analyzing rates of translation-equivalent errors in Experiment 1. Predictors significant at p < 0.05 in bold. AIC/BIC = Akaike Information Criterion/Bayesian Information Criterion.

Formula = Translation_Equivalent ~ Target_Position + Age + Trial_Order + log(Target_Frequency) + Syntactic_Grammaticality * Spanish_Profieiency + (1 + Syntactic_Grammaticality | Participant) + (1 + Syntactic_Grammaticality | Item)

Predictors	*Log OR*	*df*	*Chisq*	*p*
(Intercept)	-7.01			
Target_Position [2 vs 1]	1.55	*3*	*5.71*	0.127
Target_Position [3 vs 1]	1.32			
Target_Position [4 vs 1]	0.77			
Age	0.22	*1*	*1.24*	0.264
Trial Order	-0.41	*1*	*7.05*	0.008
Target_Frequency	1.01	*1*	*10.59*	0.001
Syntactic_Grammaticality	0.04	*1*	*0.00*	0.948
Spanish Proficiency	-0.09	*1*	*0.21*	0.648
Syntactic Grammaticality * Spanish_Proficiency	-0.02	*1*	*0.03*	0.870

	Random Effects
σ^2	3.29
τ_{00} Item	5.16
τ_{00} Participant	0.79
τ_{11} Item.Syntactic_Grammaticality1	0.76
τ_{11} Participant.Syntactic_Grammaticality1	0.00
ρ_{01} Item	0.36
ρ_{01} Participant	-1.00
N Participant	48
N Item	193
AIC / BIC	762 / 862

Table A3. Summary of mixed-effects model analyzing effects of Syntactic Grammaticality and Semantic Interpretability on target identification in Experiment 2. Predictors significant at p < 0.05 in bold. AIC/BIC = Akaike Information Criterion/Bayesian Information Criterion.

Formula = Accuracy ~ Target_Position + Age + Trial Order + log(Target_Frequency) + (Syntactic_Grammaticality + Semantic_Interpretability) * Spanish_Proficiency + Syntactic_Grammaticality:Semantic_Interpretability + (1 + Syntactic_Grammaticality + Semantic_Interpretability | Participant) + (1 + Syntactic_Grammaticality + Semantic_Interpretability | Item)

Predictors	*Log OR*	*CI*	*df*	*Chisq*	*p*
(Intercept)	-1.20	-1.51 – -0.88			
Target Position [2 vs 1]	1.83	1.45 – 2.22	*3*	*86.59*	<0.001
Target Position [3 vs 1]	1.82	1.43 – 2.19			
Target Position [4 vs 1]	1.39	0.95 – 1.82			
Age	-0.19	-0.34 – -0.05	*1*	*6.63*	0.010
Trial Order	0.06	0.02 – 0.10	*1*	*10.74*	0.001
Target Frequency	0.21	0.05 – 0.35	*1*	*7.84*	0.005
Nontarget Frequency	-0.05	-0.18 – 0.08	*1*	*0.65*	0.420
Syntactic Grammaticality	0.15	0.08 – 0.22	*1*	*17.90*	<0.001
Semantic Interpretability	0.02	-0.03 – 0.07	*1*	*0.87*	0.351
Spanish Proficiency	0.12	-0.04 – 0.26	*1*	*2.38*	0.123
Syntactic Grammaticality * Semantic Interpretability	0.03	0.003 – 0.06	*1*	*4.58*	0.032
Syntactic Grammaticality * Spanish Proficiency	0.04	0.01 – 0.08	*1*	*8.17*	0.004
Semantic Interpretability * Proficiency	0.002	-0.03 – 0.03	*1*	*0.01*	0.910

	Random Effects
σ^2	3.29
τ_{00} Item	0.83
τ_{00} Participant	0.71
τ_{11} Item.Syntactic Grammaticality	0.19
τ_{11} Item.Semantic Interpretability	0.07
τ_{11} Participant.Syntactic Grammaticality	0.00

τ_{11} Participant.Semantic Interpretability	0.00
ρ_{01}	-0.13
	-0.05
	1.00
	1.00
N Participant	135
N Item	196
Observations	26442
AIC / BIC	28923 / 29144

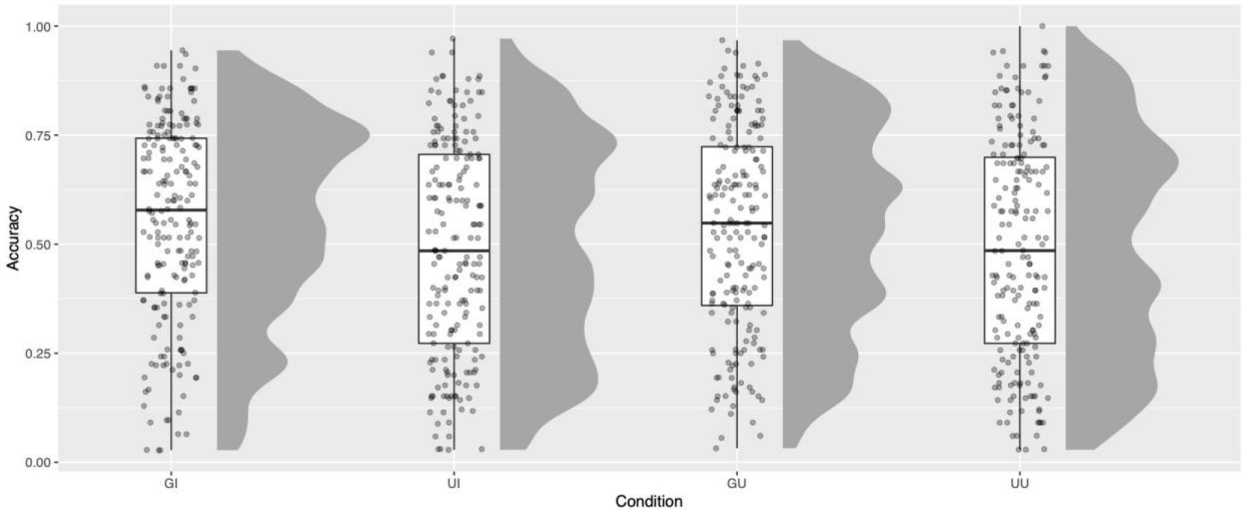

Figure A2. Results of Experiment 2, raw accuracy, by items. GI = Grammatical Syntax, Interpretable Semantics; UI = Ungrammatical Syntax, Interpretable Semantics; GU = Grammatical Syntax, Uninterpretable Semantics; UU = Ungrammatical Syntax, Uninterpretable Semantics.. The box plots depict the median and interquartile ranges, with each dot representing a single item.

References

- Bates, D., Maechler, M., Bolker, B., & Walker, S. (2015). Fitting Linear Mixed-Effects Models Using lme4. Journal of Statistical Software, 67(1), 1-48.
- Lenth, R. (2019). emmeans: Estimated Marginal Means, aka Least-Squares Means. R package version 1.4.1. https://CRAN.R-project.org/package=emmeans
- Perea, M., Baciero, A., & Marcet, A. (2021). Does a mark make a difference? Visual similarity effects with accented vowels. Psychological Research, 85(6), 2279–2290.

- R Core Team (2022). R: A language and environment for statistical computing. R Foundation for Statistical Computing, Vienna, Austria. URL https://www.R-project.org/.
- Rubin, M. (2017). Do p Values Lose Their Meaning in Exploratory Analyses? It Depends How You Define the Familywise Error Rate. Review of General Psychology, 21(3), 269–275.
- Singmann, H., Bolker, B., Westfall, J. , Aust, F., & Ben-Shachar, M. (2022). afex: Analysis of Factorial Experiments. R package version 1.1-1, https://CRAN.R-project.org/package=afex
